# Will College Members Please Note

**Published:** 1921-01-15

**Authors:** 


### Will College Members Please Note.

There are not many weeks to elapse before Parliament meets and the Hours of Employment Bill may be before it at any moment thereafter. The policy of the College will then have to be indicated at once. It is, therefore, urgent that members should without further delay send in their replies to the Referendum posted to them last month. There are thousands of College members who have not yet replied, and this failure on their part must affect the influence which the College may be able to exercise. Already the College has been asked by the Ministry of Labour the proportion of its members wishing exclusion from the Unemployment Insurance Act. The College's reply must have carried more weight had members in greater numbers sent in their answers to this question immediately on receipt of the form received. They are therefore asked to delay no longer. Their subscriptions can be sent later, if more convenient, but **their replies to the questions asked last month are wanted now.**

